# Unambiguous Determination of Benzo[a]pyrene and Dibenzo[a,l]pyrene in HPLC Fractions via Room-Temperature Fluorescence Excitation–Emission Matrices

**DOI:** 10.3390/molecules30071550

**Published:** 2025-03-31

**Authors:** George T. Knecht, Stephanie D. Nauth, Juan C. Gomez Alvarado, Anthony M. Santana, Hector C. Goicoechea, Andres D. Campiglia

**Affiliations:** 1 Department of Chemistry, University of Central Florida, Physical Sciences Building, 4111, Orlando, FL 32816, USAju916043@ucf.edu (J.C.G.A.);; 2 Laboratorio de Desarrollo Analítico y Quimiometría, Catedra de Química Analítica I, Facultad de Bioquímica y Ciencias Biológicas, Universidad Nacional del Litoral, Ciudad Universitaria, Santa Fe 3000, Argentina; 3 Consejo Nacional de Investigacioes Científicas y Técnicas (CONICET), Godoy Cruz 2290, Buenos Aires CP C1425FQB, Argentina

**Keywords:** HPLC, room-temperature fluorescence, excitation emission matrices, PARAFAC, benzo[a]pyrene, dibenzo[a,l]pyrene

## Abstract

When high-performance liquid chromatography (HPLC) is used for the analysis of polycyclic aromatic hydrocarbons (PAHs) in complex samples, further examination of HPLC fractions is recommended to confirm PAH assignments solely based on retention times. Gas chromatography–mass spectrometry (GC-MS) has been particularly relevant in the unambiguous determination of PAHs with remarkably similar retention times. The combination of HPLC and GC requires lengthy analysis times to ensure proper assignments. This article presents an approach for the analysis of co-eluted PAHs with no need for further chromatographic separation. Benzo[a]pyrene (BaP) and dibenzo[a,l]pyrene (DBalP) were directly determined in a co-eluted HPLC fraction via room-temperature fluorescence excitation–emission matrices (RTF-EEMs). RTF-EEMs can be recorded in a matter of seconds with a spectrofluorometer equipped with a multichannel detection system. The spectral overlapping of BaP and DBalP was resolved using parallel factor analysis (PARAFAC). The analytical advantages of this approach were demonstrated with the trace analysis (ng/mL) of these two PAHs in pre-concentrated tobacco extracts.

## 1. Introduction

HPLC is a popular approach to the qualitative and quantitative analysis of polycyclic aromatic compounds in many scientific fields. Commercial instrumentation for the HPLC analysis of PAHs is usually equipped with ultraviolet–visible (UV-VIS) and/or fluorescence (FL) detectors. Although multichannel detectors with on-the-fly spectral scanning capabilities are intrinsically more selective than single-channel detectors, they lack specificity for the unambiguous identification of co-eluted compounds. This is particularly true for the analysis of PAHs with similar HPLC retention behaviors and, therefore, results in almost identical retention times [1,2,3]. The unambiguous identification of co-eluted PAHs often requires further analysis of the co-eluted HPLC fractions with a supporting analytical technique such as GC-MS. Unfortunately, the combination of HPLC and GC requires costly experimental procedures with rather lengthy analysis times to ensure proper PAH assignments.

Due to their ubiquitous occurrence and extreme eco-toxicological relevance, the U.S. Environmental Protection Agency (EPA) recommends the routine monitoring of sixteen PAHs with molecular masses (MMs) ranging from 128 Da to 278 Da. These include naphthalene, acenaphthene, acenaphthylene, fluorene, phenanthrene, anthracene, fluoranthene, pyrene, benzo[*a*]anthracene, chrysene, benzo[*b*]fluoranthene, benzo[*k*]fluoranthene, BaP, dibenzo[*a*,*h*]anthracene, benzo[*g*,*h*,*i*]perylene, and indeno[1,2,3-*cd*]pyrene. According to the International Agency for Research on Cancer (IARC), BaP has the highest cancer risk among the EPA PAHs [4].

In addition to the sixteen priority pollutants, the toxicity of PAH-contaminated samples is attributed to the presence of high-molecular-weight PAHs (HMW-PAHs), i.e., PAHs with an MM greater than 300 Da. As a matter of fact, certain PAHs with an MM of 302 Da have shown higher toxicity than the sixteen EPA PAHs. These include DBalP, which is the most potent carcinogenic PAH known to date [5,6,7,8]. Its toxicity is approximately 100 times the toxicity of BaP. Thus, unambiguous identification of DBalP in contaminated samples is extremely relevant even if it is present at lower concentrations than other PAHs.

In a recent article, Campiglia and co-workers applied EPA Method 610 to the HPLC analysis of 15 EPA PAHs and 10 HMW-PAHs with an MM of 302 Da from tobacco [9]. Except for DBalP, all the 302 Da isomers showed the expected chromatographic behaviors under reversed-phase conditions, i.e., they eluted later than the EPA PAHs from the chromatographic column due to the stronger affinities for the octadecyl stationary phase [2]. DBalP co-eluted with BaP and therefore, eluted earlier than three heaviest EPA PAHs, namely dibenzo[*a*,*h*]anthracene, benzo[*g*,*h*,*i*]perylene, and indeno[1,2,3-*cd*]pyrene.

The unambiguous determination of DBalP and BaP in HPLC fractions was then accomplished via Laser-Excited Time-Resolved Shpol’skii Spectroscopy (LETRSS) [9]. The two PAHs were collected within their time-window of HPLC elution (29-32 min). The mobile phase (acetonitrile) was evaporated to dryness and the residue was reconstituted in 1mL of octane. The LETRSS analysis was carried out at 77 K with the aid of a cryogenic fiber optic probe and an instrumental set-up that was built in-house for time-resolved laser-induced fluorescence measurements. Wavelength time matrices (WTMs) were collected from the frozen samples to obtain line-narrowed spectra and fluorescence lifetimes resulting in the unambiguous determination of BaP and DBalP.

The present study proposes a straightforward approach to the analysis of these two PAHs in HPLC fractions. It consists of pouring the HPLC fraction directly into a quartz cuvette to record RTF-EEMs using a commercial spectrofluorometer. Since the spectrofluorometer is equipped with a spectrograph and a charge-coupled device (CCD), recording RT-EEMs from HPLC fractions only takes a few seconds per sample. Spectral deconvolution was accomplished with PARAFAC. Although this algorithm has been applied to the analysis of several EPA PAHs via RTF [10,11,12,13,14,15,16,17], our literature search did not find any reports of the application of PARAFAC for the unambiguous determination of BaP and DBalP in HPLC fractions. The same was true for the analysis of these two PAHs in tobacco samples. Although B[a]P has been found in a variety of tobacco products and cigarette smoke, there are no reports that detected the presence of DBalP using chromatographic methods [18,19,20,21]. This is rather intriguing, particularly if one considers the incomplete combustion of tobacco during the smoking process. The straightforward experimental procedure, the excellent analytical figures of merit, and the accuracy of the analysis presented here make this approach a robust alternative for the routine analysis of BaP and DBalP in tobacco samples.

## 2. Results and Discussion

**Figure 1:**
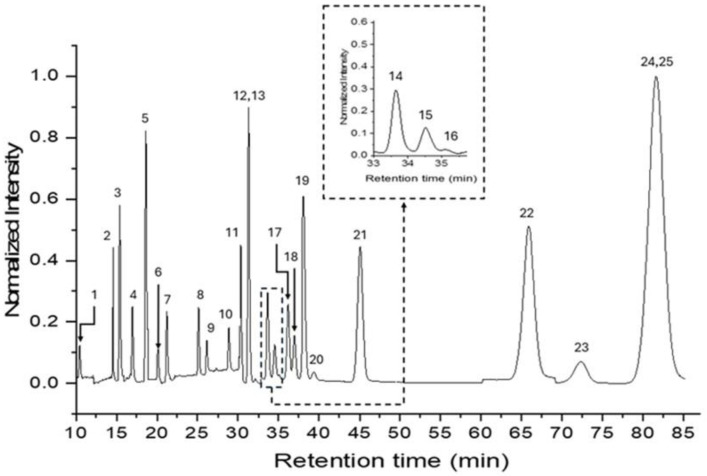
Typical HPLC chromatogram recorded from a standard solution containing the 16 EPA PAHs and 10 HMW-PAHs in acetonitrile. All PAH concentrations were at the ng/mL level. Fluorescence measurements were performed at the maximum excitation and emission wavelengths of each PAH [9].

Figure 1 shows a typical chromatogram of a standard mixture containing the 25 studied PAHs [9]. The co-elution of BaP and DBalP occurred between an elution time of approximately 30 and 32 min, and it was further confirmed by the statistical equivalence (P ≥ 98%; n_1_ = n_2_= 3) [22] of their retention times (t_R_^BaP^ = 30.9 ± 0.3 min; t_R_^DBalP^ = 31.8 ± 0.3 min). Considering the experimental simplicity of pouring the HPLC fraction into a cuvette for measurements using a spectrofluorometer, we studied the RTF features of BaP and DBalP in acetonitrile, i.e., the mobile phase of the HPLC separation.

### 2.1. Room Temperature Excitation and Fluorescence Spectra of BaP and DBalP

**Figure 2:**
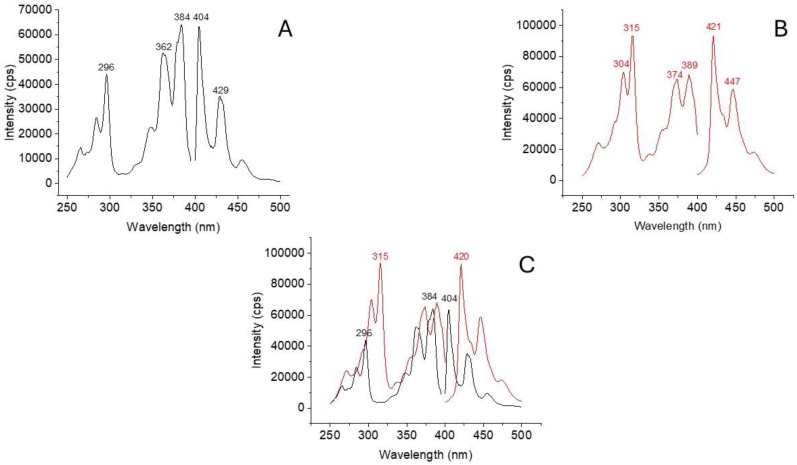
Excitation and fluorescence spectra of BaP (**A**) and DBalP (**B**) recorded from pure standard solutions in acetonitrile. The excitation and emission spectra were recorded at the maximum emission and excitation wavelengths of each PAH, respectively. The excitation and emission passbands were 3 nm in all cases. Figure (**C**) illustrates the spectral overlap of these two PAHs in acetonitrile solutions. All spectra were normalized for the maximum fluorescence intensities.

Figure 2A and 2B show the excitation and emission spectra of BaP and DBalP recorded from pure standard solutions in acetonitrile, respectively. All spectra were recorded at the maximum excitation and emission wavelengths of each PAH using a 3 nm band-pass for both monochromators. Although the two PAHs showed characteristic spectral profiles with distinct excitation and emission wavelengths, the strong spectral overlapping observed in Figure 2C might prevent their unambiguous determination in HPLC fractions. This possibility was investigated with binary mixtures of BaP and DBalP in acetonitrile. Table S1 summarizes the results obtained for BaP. The possible interference of DBalP was tested at the same concentration of BaP (1:1 mixture) and at 5 × (1:5 mixture) and 10 × (1:10 mixture) higher concentrations of DBalP compared to BaP. All fluorescence intensities were measured at the maximum excitation and emission wavelength of BaP and then compared to the fluorescence intensity of a pure standard solution of BaP. No interference from DBalP was observed for the 1:1 mixture. As shown in Table S2, similar results were obtained for DBalP in the presence of BaP.

### 2.2. Room-Temperature Fluorescence Excitation–Emission Matrices (RTF-EEMs) of BaP and DBalP

The unambiguous determination of co-eluted PAHs in HPLC fractions is limited by the broad nature of room-temperature excitation and fluorescence spectra. Well-known approaches to reducing the overlap of excitation and fluorescence spectra include coupling high-order instrumental data to parallel factor analysis (PARAFAC). This approach carries with it the second-order advantage, which permits the quantification of fluorophores in samples of unknown composition no matter how many signal-overlapping constituents are in the unknown sample [23,24,25,26,27].

Previous work in Campiglia’s lab on the analysis of EPA PAHs in soil samples combined PARAFAC with 4.2K time-resolved EEMs (4.2K TREEMs) [28]. TREEMs refer to excitation–emission matrices recorded with laser-based instrumentation at certain time windows during the total fluorescence decay of the sample. The present study combined PARAFAC and RT-EEMs, which consisted of fluorescence spectra recorded at several excitation wavelengths and compiled into either a two-dimensional data format (wavelength of excitation vs. wavelength of fluorescence) or a three-dimensional format (wavelength of excitation vs. wavelength of fluorescence vs. signal intensity).

**Figure 3:**
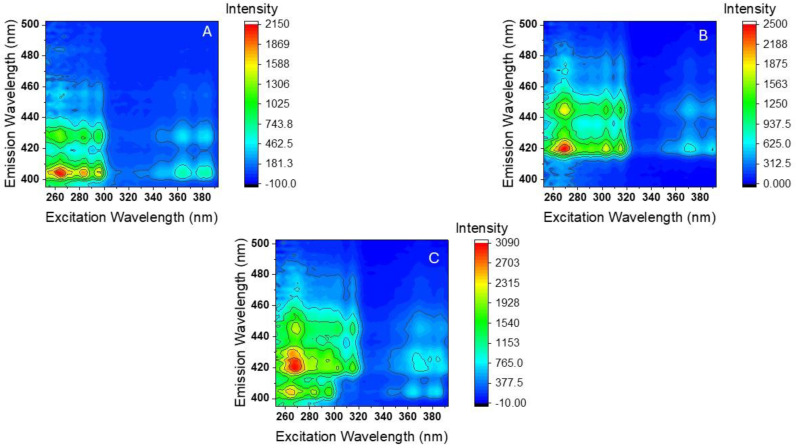
RTF-EEMs recorded from pure standard solutions of (**A**) BaP and (**B**) DBalP in acetonitrile and (**C**) a binary mixture of BaP and DBalP in acetonitrile. In all cases, the concentrations of BaP and DBalP were 29 ng/mL and 281 ng/mL, respectively. All measurements were performed using 3 nm excitation and emission passbands.

Figure 3A,B show the RT-EEMs recorded from pure standard solutions of BaP and DBalP in acetonitrile. Figure 3C shows the RTF-EEM recorded from a binary mixture of the two PAHs in acetonitrile. The excitation and emission passbands were set at 3 nm in all cases. The sample excitation was conducted at 3 nm increments between 250 and 393 nm. The excitation wavelengths were scanned from low (393 nm) to high energy (250 nm) to reduce the sample exposure to UV irradiation and possible photobleaching. The fluorescence emission was monitored between 394 and 502 nm using an integration time of 150 milliseconds. Scatter interference from excitation radiation and from second-order grating effects were eliminated with the aid of fully automated cut-off filters. Under these instrumental settings, each EEM resulted in a data matrix of 55 excitation data points × 48 emission data points, with an average acquisition time of 20.7 ± 0.3 s per EEM.

### 2.3. RTF-EEMs’ Analytical Figures of Merit for the Analysis of BaP and DBalP in Acetonitrile

The analytical figures of merit (AFOM) for the analysis of BaP and DBalP in acetonitrile are summarized in Table 1. The fluorescence intensities plotted in the calibration graphs correspond to the intensity values obtained from the EEMs at the maximum excitation and emission wavelengths of each PAH. No attempts were made to experimentally determine the upper concentration limits of the linear dynamic ranges (LDRs). The correlation coefficients (Rs) were close to unity, demonstrating linear correlations between fluorescence intensity and PAH concentration. The LOD and the LOQ of BaP were approximately one order of magnitude lower than those of DBalP. These values are in good agreement with the stronger fluorescence emitted by BaP. The relative standard deviations (RSDs) at medium linear concentrations demonstrated good reproducibility at the parts-per-billion (ng/mL) concentration level.

### 2.4. Calibration and Validation Sets for PARAFAC Analysis

If EEMs are arranged in a three-dimensional representation ***X*** of dimensions *I* × *J* × *K*, where *I* is the number of samples, *J* is the number of emission wavelengths, and *K* is the number of excitation wavelengths, PARAFAC can decompose the array into a more condensed form than the original one [23]. This is accomplished by minimizing the sum of the squares of the residuals (*e_ijk_*) in the model described by Equation (1):

(1) $$x_{ijk}=\sum_{n=1}^{n}a_{in}b_{jn}c_{kn}+e_{ijk}$$

where *n* indicates the component number and *x_ijk_* is the fluorescence intensity for sample *i* at the emission wavelength *j* and excitation wavelength *k.* For any given component *n*, the elements *a_in_*, *b_jn_*, and *c_kn_* are arranged in the score vector **a***_n_* (whose elements are directly proportional to its concentration in each sample) and the loading vectors **b***_n_* and **c***_n_*, which estimate its emission and excitation profiles.

Binary mixtures with different concentrations of BaP and DBalP were prepared in acetonitrile for the calibration and the validation sets. Three individual EEMs were recorded from three aliquots of each binary mixture to account for possible instrumental variations. The same approach was used to record EEMs from the blank solution (acetonitrile). The average plots of the blank-subtracted EEMs recorded from all the binary mixtures in the calibration set and the validation set are shown in Figure S1 and Figure S2 respectively.

Table 2 summarizes the composition of the calibration set for the PARAFAC model employed in these studies. All solutions consisted of standard mixtures of BaP and DBalP in acetonitrile. The concentrations of the two PAHs were calculated with a Central Composite Design (CCD) and took into consideration the LDRs in Table 1.

Because BaP is a stronger fluorophore than DBalP, its concentration in all the binary mixtures was considerably lower than the concentration of DBalP. In addition, a zero-concentration sample for each analyte was included in the calibration sets. Table 3 lists the nominal concentrations of BaP and DBalP in the binary mixtures for the validation set and those predicted by PARAFAC using two factors. For the binary mixtures, there was no need to exploit the second-order advantage. The nominal concentrations of the standard mixtures were different from those in Table 1 but within the same concentration ranges as the calibration set. A comparison of the nominal to the predicted concentration clearly showed an excellent predictive ability, with mean recoveries of 102.2 ± 5% for BaP and 100.4 ± 3% for DBalP.

### 2.5. HPLC–EEM Analysis of Tobacco Smoke Condensate

**Figure 4:**
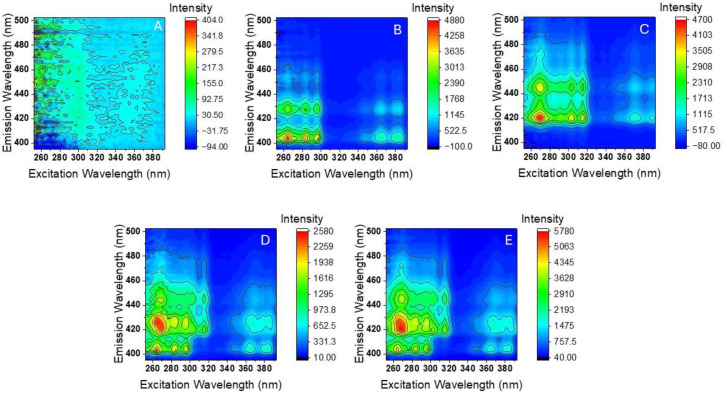
RTF-EEMs of HPLC fractions recorded from TSC mixed with different concentrations of BaP and DBalP (**B**–**E**) and from unmixed TSC (**A**).

Five samples of tobacco smoke condensate (TSC) were subjected to HPLC analysis. Four samples were prepared by mixing aliquots of pre-concentrated TSC to solid amounts of BaP and DBalP. The remaining sample consisted of TSC with no addition of BaP and DBalP. Figure 4 shows the RTF-EEMs recorded from the five HPLC samples.

A visual comparison of the EEMs in Figure 3 provides insights into the contribution of each PAH to the total fluorescence of the HPLC fractions. The nominal concentrations of BaP and DBalP in the five TSC samples are listed in Table 4. It should be noted that the reported concentrations only compute the amounts of BaP and DBalP added to the TSC samples. The excellent agreement between the nominal and the predicted concentration demonstrate the ability of PARAFAC to determine the two PAHs in co-eluted HPLC fractions. These quantitative results are consistent with the quality of the profiles extracted by PARAFAC for both analytes (see SI Figure 3).

## 3. Materials and Methods

### 3.1. Chemicals

All reagents used were the highest available purity. Analytical standards of BaP and DBalP were purchased from Chiron (Trondheim, Norway). Pure (100%) HPLC-grade acetonitrile and acetone were purchased from Fisher Scientific (Waltham, MA, USA). Camel Menthol Crush commercial cigarettes were purchased at local stores. Nanopure water supplied by the Barnstead Nanopure Infinity water system (Barnstead, Dubuque, IA, USA) located in the laboratory was used throughout all the experiments.

### 3.2. Preparation of Calibration and Validation Sets for PARAFAC Analysis

Calibration and validation samples were prepared by diluting stock solutions of BaP and DBalP in HPLC-grade acetonitrile. All dilutions were made with micropipettes to a total volume of 3.00 mL. The PAH final concentrations varied between 16.0 ng/mL and 104.0 ng/mL for BaP, and between 164.6 ng/mL and 500.0 ng/mL for DBalP. In addition, a zero-concentration sample for each analyte was included in the calibration sets. For the validation sets, nine solutions were prepared with concentrations varying from 30.0 ng/mL to 95.0 ng/mL for BaP and from 110 ng/mL to 450 ng/mL for DBalP.

### 3.3. Collection of Tobacco Smoke Condensate

Tobacco smoke condensate was collected according to the guidelines of the National Institute of Standards and Technology (NIST SRM 3222) [29]. The main steps were as follows: A sintered glass frit funnel was placed on an Erlenmeyer vacuum flask with a length of tubing extending from the funnel into 300 mL of acetone. The apparatus was placed in dry ice and a slight vacuum was created. After setup, 11 g of cigarette tobacco filler was ignited and burned to ash in the filter, such that the smoke bubbled through the acetone. After 2 min, the ashes were stoked, and the apparatus allowed to cool for 8 min. After cooling, the remaining ashes were removed, and the funnel was washed with around 200 mL of acetone. The funnel and tubing were then removed and sonicated in the extract solution to remove any remaining condensate.

### 3.4. HPLC–EEM Analysis of Tobacco Smoke Condensate

Prior to HPLC analysis, a pre-concentration step for the tobacco smoke condensate was performed by evaporating 8 mL of the condensate to a residue, which was then reconstituted with acetonitrile to a final volume of 1.0 mL and centrifuged for 1 min at 10,000 rpm. Samples for PARAFAC analysis were prepared by adding 100 μL aliquots of the pre-concentrated smoke condensate to solid residues containing various masses of either BaP or DBalP. BaP residues were prepared by evaporating different volumes of a 101.2 mg/mL BaP standard solution in acetonitrile. DBalP residues were prepared by evaporating different volumes of a 513.0 mg/mL DBalP standard solution in acetonitrile. All mixtures were centrifuged at 600 rpm for 5 min to ensure complete dissolution of the solid residue into the liquid aliquot of the pre-concentrated smoke condensate. All sample injections were performed using a 20 μL fixed-volume injection loop. HPLC fractions were collected from 27.5 to 31.5 min of the chromatographic time and subjected to EEM analysis. Each HPLC fraction was subjected to triplicate measurements made from three sample injections.

### 3.5. Instrumentation

Chromatographic analysis was performed with a Hitachi HPLC system (San Jose, CA, USA) using a computer with Hitachi software. Its main components included a model L7100 gradient pump, an L-7400 UV detector, an L-7485 fluorescence detector, an L-761 online degasser, and a D-7000 control interface. PAH separation was accomplished with an Eclipse PAH column (250 mm length, 4.6 mm inner diameter, and 5 µm particle size), using isocratic elution for five minutes with 40% acetonitrile and 60% water, and then linear gradient elution to 100% with acetonitrile over 25 min. The mobile phase flow rate was 2.0 mL/min and the column temperature was ~25 °C. Sample injection (20 µL) was performed using a fixed-volume injection loop. HPLC fractions were collected in 30 mL amber vials.

Room-temperature fluorescence measurements were made using a Duetta^TM^ spectrofluorometer (Horiba Scientific, Piscataway, NJ, USA) equipped with a 75 W xenon arc lamp, a single-grating scanning monochromator for sample excitation, and a spectrograph/TE-cooled CCD for spectra collection from 250 nm to 1100 nm. The instrument is equipped with a reference detector (Si photodiode) to compensate for variations in the intensity of sample excitation and a fully automated set of cut-off filters to eliminate second-order grating effects. Instrument control was accomplished with custom software (EzSpec^TM^ Touch-Screen Software, https://www.horiba.com/jpn/scientific/products/detail/action/show/Product/ezspec-software-1880/#show-more). All measurements were made from liquid solutions in 500 μL quartz cuvettes.

### 3.6. Software for Data Analysis with PARAFAC

MATLAB 7.10 was used for all calculations (The Mathworks Inc., Natick, MA, USA, 2010) [30]. Software processing was facilitated by an MVC2 graphical user interface written in MATLAB that was previously described by Olivieri et al. [31].

## 4. Conclusions

Although DBalP is the most toxic PAH known to date, there is relatively limited information on its possible presence in tobacco samples. Our study showed that DBalP co-elutes with BaP under standard HPLC conditions. Therefore, their unambiguous identification requires further analysis of the HPLC fractions via an alternative analytical method. One possibility is to perform the analysis via GC-MS. This technique is able to differentiate BaP from DBalP in HPLC fractions, but it would require further separation via a rather lengthy experimental procedure.

Herein, an alternative method was proposed to obviate further chromatographic separation of these two PAHs in co-eluted HPLC fractions. The experimental procedure is straightforward; it consists of pouring the HPLC fraction directly into a quartz cuvette to record RTF-EEMs using a commercial spectrofluorometer. If the spectrofluorometer is equipped with a multichannel detection system, i.e., a spectrograph and a CCD, the RTF-EEM recording will take less than one minute per HPLC fraction. These are distinct advantages compared to LETRSS analysis [9], which requires laser-based instrumentation and the use of a liquid cryogen to avoid spectral overlap. In the present study, the strong spectral overlap between BaP and DBalP was resolved at room temperature with the aid of PARAFAC. Since this algorithm carries with it the second-order advantage, it makes recording RTF-EEMs an accurate approach for the unambiguous determination of BaP and DBalP at the parts-per-billion level via EPA Method 610.

## Figures and Tables

**Table 1:** RTF-EEMs’ analytical figures of merit for BaP and DBalP in acetonitrile.

	λ_exc_/λ_em_ ^1^	LOD ^2^ (ng/mL)	LOQ ^3^ (ng/mL)	LDR ^4^ (ng/mL)	R ^5^	% RSD ^6^
BaP	384/404	4.7	16	16–104	0.9995	0.6
DBalP	315/421	32	107	107–500	0.9988	4.2

^1^ Excitation (λ_exc_) and emission (λ_em_) wavelengths used for fluorescence measurements. ^2^ Limit of detection (LOD) was calculated using LOD = 3S_B_/m, where S_B_ is the standard deviation of sixteen blank signal measurements and m is the slope of the calibration curve. ^3^ Limit of quantitation (LOQ) was calculated using LOQ = 10S_B_/m. ^4^ Linear Dynamic Range (LDR), in ng/mL, extends from the LOQ to an arbitrarily chosen upper linear concentration. ^5^ Correlation coefficient (R) of LDR. ^6^ Relative standard deviation (RSD) = S/I × 100, where I is the average intensity and S is the standard deviation of the intensity calculated from three measurements at medium linear concentrations.

**Table 2:** Concentrations of BaP and DBalP in binary mixtures employed for PARAFAC calibration.

Sample	BaP (ng/mL)	DBalP (ng/mL)
1	28.9	164.6
2	91.1	164.6
3	28.9	442.4
4	91.1	442.4
5	16.0	303.5
6	104.0	303.5
7	60.0	107.0
8	60.0	500.0
9	60.0	303.5
10	0.0	303.5
11	60.0	0.0

**Table 3:** Concentrations of BaP and DBalP in binary mixtures employed for PARAFAC validation.

Sample	BaP (ng/mL)		DBalP (ng/mL)	
	Nominal ^1^	Predicted ^2^	Nominal ^1^	Predicted ^2^
1	39.5	37.8	159.8	165.0
2	85.5	87.9	159.8	156.9
3	39.5	40.9	400.2	382.9
4	85.5	89.9	400.2	431.5
5	30.0	32.6	280.0	267.2
6	95.0	87.9	280.0	277.2
7	62.5	64.1	110.0	117.2
8	62.5	65.7	450.0	428.4
9	62.5	64.3	280.0	286.9

^1^ Nominal concentration refer to the actual concentrations of BaP and DBalP in the binary mixture. ^2^ Predicted concentrations refer to the concentrations calculated by PARAFAC.

**Table 4:** PARAFAC predictions of BaP and DBalP concentrations spiked into samples of tobacco.

Sample	BaP (ng/mL)		DBalP (ng/mL)	
	Nominal ^1^	Predicted ^2^	Nominal ^1^	Predicted ^2^
1	0.0	–	0.0	–
2	85.5	88.4	0.0	–
3	0.0	–	400.2	387.2
4	39.5	38.3	159.8	162.1
5	85.5	87.9	400.2	406.1
Average Recovery (%)	–	100.9	–	99.9

^1^ Nominal concentration refer to the actual concentrations of BaP and DBalP in the binary mixture. ^2^ Predicted concentrations refer to the concentrations calculated by PARAFAC.

## Data Availability

Data are contained within the article and Supplementary Materials.

## Supplementary Materials

The following supporting information can be downloaded at: https://www.mdpi.com/article/10.3390/molecules30071550/s1, Table S1: Statistical comparison of fluorescence intensities recorded at the maximum excitation and emission wavelengths of BaP in 1:1, 1:5, and 1:10 binary mixtures with DBalP in acetonitrile; Table S2: Statistical comparison of fluorescence intensities recorded at the maximum excitation and emission wavelengths of DBalP in 1:1, 1:5, and 1:10 binary mixtures with BaP in acetonitrile; Figure S1: RTF-EEMs recorded from the calibration set used for the PARAFAC analysis; Figure S2: RTF-EEMs recorded from the validation set used for the PARAFAC analysis; Figure S3: Examples of spectral profiles extracted by PARAFAC from HPLC fractions.
