# Supplementary material for: Unambiguous Determination of Benzo[a]pyrene and Dibenzo[a,l]pyrene in HPLC Fractions via Room-Temperature Fluorescence Excitation–Emission Matrices
Source: Molecules. 2025 Mar 31;30(7):1550. doi: 10.3390/molecules30071550 (PMC11990361; doi:10.3390/molecules30071550)
Supplement: Supplementary file 1 [file molecules-30-01550-s001.zip › molecules-3560630-supplementary.pdf]

Unambiguous Determination of Benzo[a]pyrene and Dibenzo[a,l]pyrene in HPLC Fractions via Room-Temperature Fluorescence Excitation–Emission Matrices

George T. Knecht [1], Stephanie D. Nauth [1], Juan C. Gomez Alvarado [1], Anthony M. Santana [1], Hector C. Goicoechea [2,3] and Andres D. Campiglia [1,*]

[1] Department of Chemistry, University of Central Florida, Physical Sciences Building. 4111, Orlando, FL 32816, USA
[2] Laboratorio de Desarrollo Analítico y Quimiometría, Catedra de Química Analítica I, Facultad de Bioquímica y Ciencias Biológicas, Universidad Nacional del Litoral, Ciudad Universitaria, 3000, Santa Fe, Argentina
[3] Consejo Nacional de Investigacioes Científicas y Técnicas (CONICET), Godoy Cruz 2290 CP C1425FQB, Buenos Aires, Argentina
* Correspondence: andres.campiglia@ucf.edu

Table S1. RTF Analysis of BaP in the Presence of DBalP using Acetonitrile as the Solvent

Mixture BaP:DBalP	I_{mix} (cps) [a]	I_{std} (cps) [b]	t_{exp} [c]	t_{crit} [d]	Statistically Equivalent?
1:1	62270 ± 780	63103 ± 790	0.520	2.776	Yes
1:5	64820 ± 509	63103 ± 790	3.130	2.776	No
1:10	66120 ± 676	63103 ± 790	5.020	2.776	No

[a] Fluorescence intensity of synthetic mixture. Average and standard deviation of three independent measurements.

[b] Fluorescence intensity of pure standard solution. Average and standard deviation of three independent measurements.

[c] t_{exp} calculated with the formula $t = \frac{\bar{X}_1 - \bar{X}_2}{s_p \cdot \sqrt{\frac{1}{n_1} + \frac{1}{n_2}}}$, where $s_p = \sqrt{\frac{(n_1-1)s^2{}_{X_1} + (n_2-1)s^2{}_{X_2}}{n_1+n_2-2}}$ [22].

[d] t_{crit} (P = 95% and 4 degrees of freedom).

Table S2. RTF Analysis of DBalP in the Presence of BaP using Acetonitrile as the Solvent

Mixture DBalP:BaP	I_{mix} (cps) [a]	I_{std} (cps) [b]	t_{exp} [c]	t_{crit} [d]	Statistically Equivalent?
1:1	91027 ± 185	93140 ± 2508	0.520	1.455	Yes
1:5	120716 ± 2756	93140 ± 2508	3.130	12.818	No
1:10	164056 ± 1395	93140 ± 2508	5.020	42.801	No

[a] Fluorescence intensity of synthetic mixture. Average and standard deviation of three independent measurements.

[b] Fluorescence intensity of pure standard solution. Average and standard deviation of three independent measurements.

[c] T_{exp} calculated with the formula $t = \frac{\bar{X}_1 - \bar{X}_2}{s_p \cdot \sqrt{\frac{1}{n_1} + \frac{1}{n_2}}}$, where $s_p = \sqrt{\frac{(n_1-1)s^2{}_{X_1} + (n_2-1)s^2{}_{X_2}}{n_1+n_2-2}}$ [22].

[d] T_{crit} (P = 95% and 4 degrees of freedom).

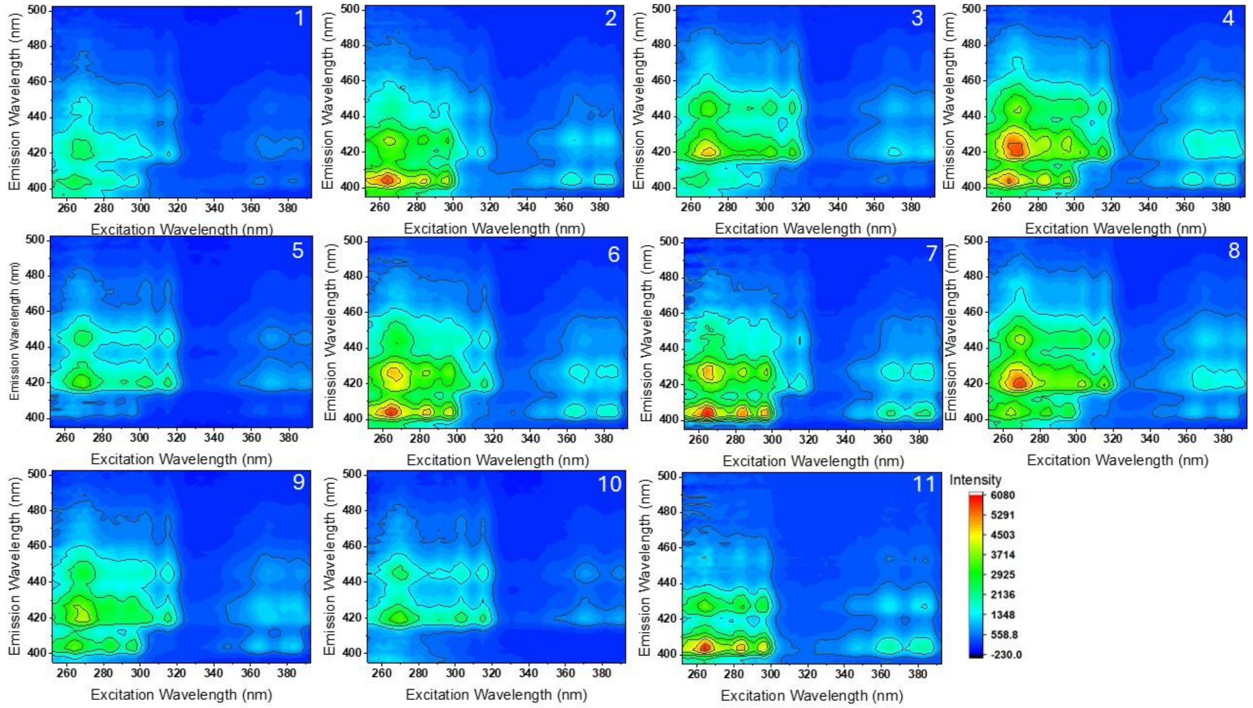

Figure S1. RTF-EEMs recorded from the calibration set used for PARAFAC analysis. Concentrations of BaP and DBalP are listed in Table 2.

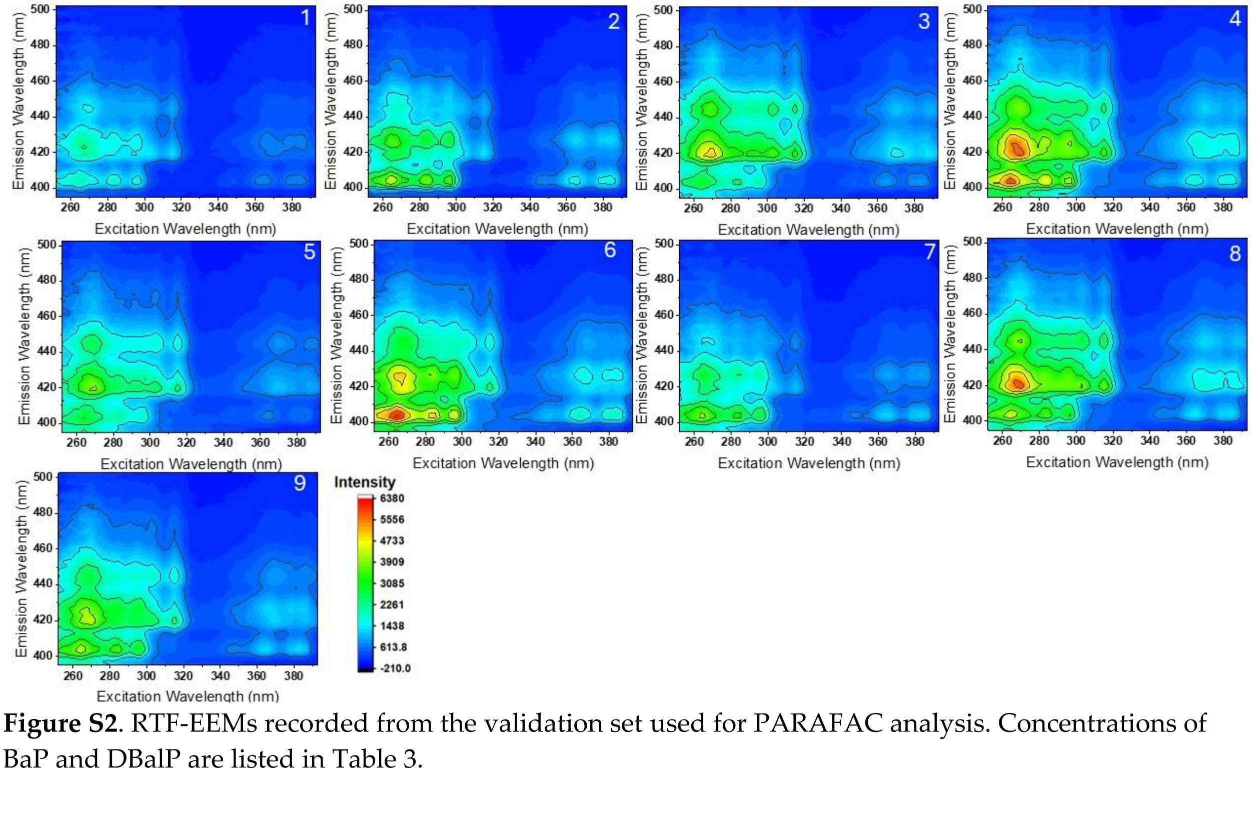

Figure S2. RTF-EEMs recorded from the validation set used for PARAFAC analysis. Concentrations of BaP and DBalP are listed in Table 3.

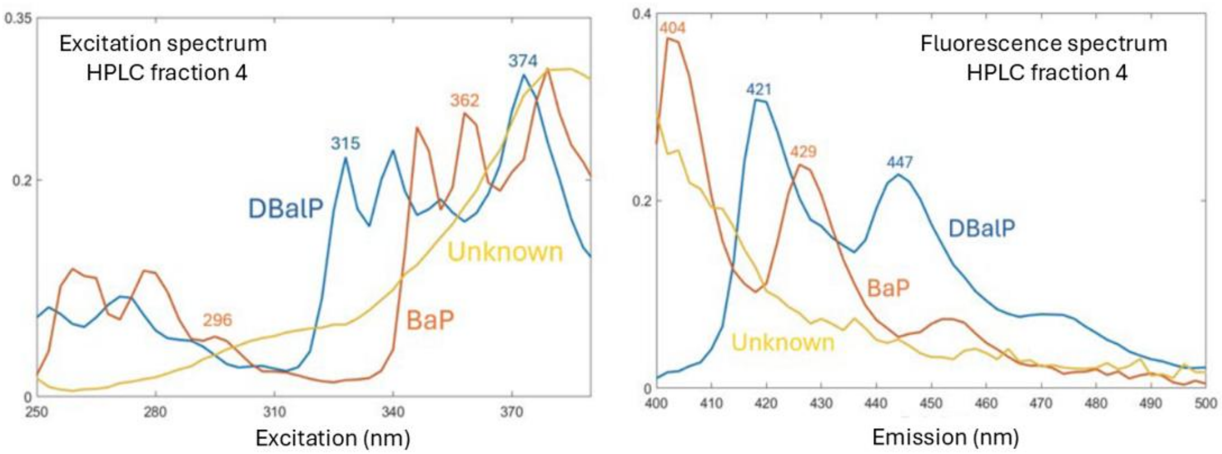

Figure S3. Examples of spectral profiles extracted by PARAFAC from HPLC fractions.
